# MicroRNA-381—A Key Transcriptional Regulator: Its Biological Function and Clinical Application Prospects in Cancer

**DOI:** 10.3389/fonc.2020.535665

**Published:** 2020-11-26

**Authors:** Xue Zeng, Zhe Cao, Wenhao Luo, Lianfang Zheng, Taiping Zhang

**Affiliations:** ^1^Department of General Surgery, Peking Union Medical College Hospital, Peking Union Medical College, Chinese Academy of Medical Sciences, Beijing, China; ^2^School of Medicine, Tsinghua University, Beijing, China; ^3^Department of Nuclear Medicine, Peking Union Medical College Hospital, Peking Union Medical College, Chinese Academy of Medical Sciences, Beijing, China; ^4^Clinical Immunology Center, Chinese Academy of Medical Sciences, Beijing, China

**Keywords:** miR-381, cancer, mechanisms of resistance, chemotherapy, biomarker

## Abstract

MicroRNAs (miRNAs) are small non-coding RNA molecules that function by regulating messenger RNAs. Recent studies have shown that miRNAs play important roles in multiple processes of cancer development. MiR-381 is one of the most important miRNAs in cancer progression. MiR-381 is downregulated in some cancers and upregulated in other cancers, including glioma, epithelial sarcoma, and osteosarcoma. MiR-381 regulates epithelial–mesenchymal transition (EMT), chemotherapeutic resistance, radioresistance, and immune responses. Thus, miR-381 participates in tumor initiation, progression, and metastasis. Moreover, miR-381 functions in various oncogenic pathways, including the Wnt/β-catenin, AKT, and p53 pathways. Clinical studies have shown that miR-381 could be considered a biomarker or a novel prognostic factor. Here, we summarize the present studies on the role of miR-381 in cancer development, including its biogenesis and various affected signaling pathways, and its clinical application prospects. MiR-381 expression is associated with tumor stage and survival time, making miR-381 a novel prognostic factor.

## Introduction

### Biogenesis of miR-381 and the Role of miR-381 in Cancer

**FIGURE 1:**
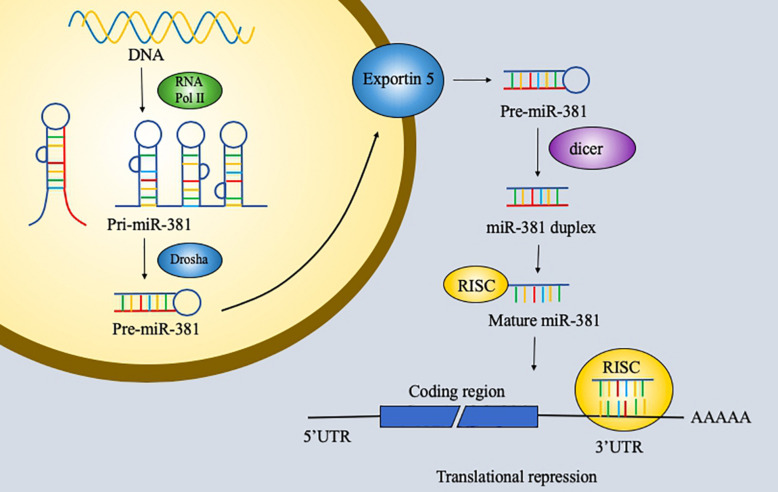
The generation of microRNA (miR)-381 in cancer.

RNA has been explored extensively and found to have notable versatility. RNA with protein-coding ability, which accounts for 1.5% of the genome, is the most commonly studied type of RNA. However, non-coding RNA is also important and remains to be explored further. Non-coding RNAs are usually classified into three groups: short non-coding RNAs, midsize non-coding RNAs, and long non-coding RNAs. MicroRNAs (miRNAs), which are 19–25 bp long, and piwi-interacting RNAs (piRNAs), which are 26–31 bp long, are considered short non-coding RNAs. MiRNAs are small endogenous non-coding single-stranded RNAs with 19–25 nucleotides and act on RNAs by degrading messenger RNAs (mRNAs) or by inhibiting their translation (1, 2). First, miRNA genes are transcribed by RNA polymerase II into 70-nucleotide-long primary transcripts that can fold into a hairpin structure (pre-miRNAs). Pre-miRNAs are processed by the Ran-GTP-dependent transporter exportin 5 and enter the cell cytoplasm. Second, the RNase III enzyme dicer cleaves the pre-miRNA. One strand of the duplex is incorporated into the miRNA-induced silencing complex (miRISC) to function. The functions of miRNA were first discovered 20 years ago. MiRNAs perform their functions via the miRISC, and in animals, miRNAs regulate gene expression by pairing to a sequence of two to eight nucleotides in the 3′ untranslated region (3′UTR) of a target mRNA to silence its expression (3, 4) (Figure 1). Furthermore, miRNAs can be involved in cell proliferation, cell differentiation, apoptosis, and cell death. The potential roles of miRNAs in tumors have been discovered and studied in the past few years. Recent studies have identified various functions of miRNAs in cancer.

**FIGURE 2:**
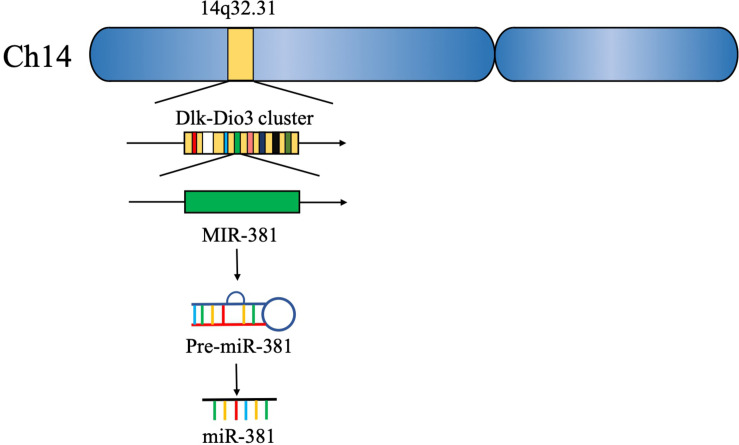
miR-381 belongs to the 14q32.31 gene cluster.

Approximately 15 years ago, 46 potential miRNA genes located in the human imprinted 14q32 domain were identified through a computer-assisted approach; 40 of these genes existed in a large cluster. The 14q32 domain, also called Dlk-Dio3 in humans and Dlk1-Gtl2 in mice, contains a large cluster of 42 miRNAs located within 10 kb of each other. MiR-381, located at the 14q32.31 locus, plays a critical role in the carcinogenesis and progression of various cancers (5, 6) (Figure 2). The expression of miR-381 is downregulated in lung adenocarcinoma, epithelial ovarian cancer, colon cancer, and breast cancer, suggesting that miR-381 may play a role as a tumor suppressor. On the other hand, miR-381 is upregulated in glioma, epithelial sarcoma, and osteosarcoma, suggesting that miR-381 might act as an oncogene. However, the mechanism of miR-381 is incompletely defined. MiR-381 plays various roles in cancer, including regulating epithelial–mesenchymal transition (EMT), the cell cycle, drug resistance, etc., resulting in tumor initiation, progression, and metastasis. These findings emphasize the importance of miR-381 in tumorigenesis and progression, and suggest that miRNA-381 may be a novel target for anticancer treatment.

## Role of miR-381 in Oncogenic Signaling Pathways

miR-381 plays multiple roles in cancers. Oncogenes have been a popular topic in cancer initiation research, and miR-381 participates in multiple oncogenic pathways. In most cancers, miR-381 acts as a tumor suppressor.

MiR-381 is downregulated in triple-negative breast cancer, which is negative for estrogen receptor (ER), progesterone receptor (PR), and human epidermal growth factor receptor 2 (HER-2) expression. In breast cancer cell lines, overexpression of miR-381 is associated with decreased levels of Wnt axis genes, including CTNNB1, RhoA, ROCK1, and c-MYC. In a mouse model, increasing miR-381 expression decreased tumor invasion and metastatic ability (7).

MiR-381 is downregulated in endometrial carcinoma tissues compared with normal endometrial tissues, and overexpression of miR-381 in endometrial carcinoma cell lines inhibits proliferation and invasion. Mechanistically, miR-381 targets IGF-1R, a key oncogene regulating cell growth, apoptosis, migration, and invasion (8, 9). Knockdown of IGF-1R reduces the tumorigenic ability of endometrial carcinoma cells, and upregulation of IGF-1R counteracts the inhibitory effects of miR-381. IGF-1R is involved in the AKT and ERK signaling pathways (10), suggesting that miR-381 suppresses tumor growth in endometrial carcinoma by directly targeting IGF-1R to modulate the AKT and ERK pathways (11).

Nicotinamide phosphoribosyltransferase (NAMPT) is an enzyme involved in the nicotinamide adenine dinucleotide (NAD) salvage pathway. NAMPT can catalyze reactions in the salvage pathway to produce NAD, which contributes to various biological processes, including DNA repair, oncogenic pathway activity, and cell cycle control. NAMPT is overexpressed in various cancers, including breast cancer, colon cancer, and gastric cancer (GC). In breast cancer cell lines, miR-381 expression is downregulated, and NAMPT protein expression is upregulated. MiR-381 suppresses NAMPT expression by binding to its 3′UTR. Transfection of an miR-381 mimic resulted in a decrease in NAD in cell lines, in turn, decreasing cell proliferation. Thus, miR-381 could be a therapeutic target in breast cancer (12).

In pancreatic cancer tissues, miR-381 is downregulated. In pancreatic cancer cell lines, overexpression of miR-381 results in reduced proliferation and invasion. In addition, the proportion of G1 phase cells is increased. In a xenograft mouse model, miR-381 inhibited tumor growth. ETS1 is targeted by miR-381 and has been found to be involved in tumorigenesis in cervical cancer, breast cancer, and gastric cancer. MiR-381 also modulates cell proliferation via the PI3K/AKT/mTOR pathway, which plays a crucial role in cell proliferation, invasion, and survival. MiR-381 acts as a tumor suppressor via inhibiting the ETS1/PI3K/AKT/mTOR pathway activity (13).

In lung adenocarcinoma samples, miR-381 is decreased. In lung adenocarcinoma cell lines, overexpression of miR-381 suppresses cell proliferation, migration, and invasion. In contrast, inhibition of miR-381 can enhance cell migration and invasion. LMO3 (LIM-only protein), which is a member of LIM-only proteins, plays an important role in cancer invasion and metastasis. Mechanistically, miR-381 regulates the PI3K/AKT pathway via inhibiting LMO3, and overexpression of miR-381 decreases the protein levels of p-PI3K and p-AKT in cell lines. By inhibiting PI3K/AKT pathway activity, miR-381 could be a tumor suppressor (9).

Pituitary tumor-transforming gene (PTTG)-1 has been identified as an oncogene in various tumors, including lung, colorectal, and liver tumors. PTTG1 contributes to angiogenesis through transactivation of fibroblast growth factor (FGF)-2 and vascular endothelial growth factor (VEGF). PTTG1 is upregulated in pituitary tumors; in contrast, the levels of microRNA-655, miR-300, miR-381, and miR-329 are decreased in pituitary tumor tissues. Overexpression of microRNA-655, miR-300, miR-381, and miR-329 decreases the viability and proliferation of human pituitary cells. In a xenograft mouse model, microRNA-655, miR-300, miR-381, and miR-329 inhibited tumor growth and PTTG1 expression. MicroRNA-655, miR-300, miR-381, and miR-329 target PTTG1 mRNA and inhibit PTTG1 expression. p53, which is one of the most important tumor suppressor genes and is mutated in a variety of human cancers, can bind to the promoters of microRNA-655, miR-300, miR-381, and miR-329, activating their transcription. P53 binds to the promoter of miR-381, leading to activation of its transcriptional activity. The p53/microRNA/PTTG1 pathway may thus be a potential target in pituitary tumors (14).

The expression of miR-381 is downregulated in epithelial ovarian cancer tissues and cell lines. Yin Yang 1 (YY1) is a transcription factor that plays an essential role in embryogenesis. Binding of YY1 to DNA can lead to activation or repression of various genes, including c-Myc, β-actin, and IFN-γ. YY1 has been shown to repress p53 signaling to determine cell fate. In addition, YY1 has been shown to promote Wnt signaling pathway activity in colorectal cancer. In epithelial ovarian cancer tissues and cell lines, miR-381 expression is decreased, and overexpression of miR-381 significantly inhibits epithelial ovarian cancer cell proliferation, migration, and invasion. YY1 is a direct target of miR-381, and YY1 downregulation results in inhibition of cell proliferation, migration, and invasion. Moreover, P53 and the Wnt signaling pathway are associated with miR-381. MiR-381 regulates the behavior of epithelial ovarian cancer cells through the miR-381–YY1–p53 and miR-381–YY1–Wnt signaling axes (15).

MiR-381 expression is also downregulated in gastric cancer tissue samples. In gastric cancer cell lines, inhibition of miR-381 enhances cell proliferation, invasion, and migration. Mechanistically, miR-381 can target the 3′UTR of ROCK2, which plays an important role in actin cytoskeleton formation, cell proliferation, and cell migration, and inhibit its expression. Thus, via ROCK2, miR-381 can act as a tumor suppressor (16).

However, in some types of cancers, miR-381 acts as an oncogene.

Gergo et al., identified another pathway, in which miR-381 targets SMARCB1, which encodes a subunit of the SWI/SNF ATP-dependent chromatin-remodeling complex. SMARCB1 expression is absent in 83% of epithelioid sarcomas, and inactivation of SMARCB1 results in aggressive tumorigenesis. Overexpression of miR-381 in cell lines can suppress SMARCB1. Under these conditions, miR-381 expression is increased in epithelioid sarcoma and acts as an oncogene via inactivation of SMARCB1 (17, 18).

In primary gliomas, the levels of miR-381 and miR-182 are increased. Expression of LRRC4, located on chromosome 7q31-32, is highly specific to the human brain. In a mouse study, LRRC4 contributed to brain development and glial cell differentiation. Therefore, LRRC4 is considered crucial for brain development. A previous study showed that LRRC4 is a tumor suppressor gene in head and neck cancers. The expression of LRRC4, which is targeted by both miR-381 and miR-182, is decreased in glioma cells. Inhibition of miR-381 and miR-182 leads to decreased cell proliferation and induces cell cycle arrest in glioma cell lines. MiR-381 regulates LRRC4, leading to upregulation of the MEK/ERK and AKT signaling and enhanced the PI3-K/AKT signaling pathway activity, in turn resulting in increased proliferation of glioma cells. The MEK/ERK and PI3-K/AKT pathways are two major cell survival pathways and play an important role in tumorigenesis, migration, and invasion. The interaction of hsa-miR-381 with LRRC4 is involved in the pathogenesis of glioma and could be a potential therapeutic target (19, 20).

Above all, oncogenic pathways play an important role in tumorigenesis. MiR-381 is involved in the Wnt/β-catenin, AKT, ERK, PI3K/AKT/mTOR, and p53 oncogenic pathways. Indeed, the role of miR-381 is complicated. In breast cancer, endometrial carcinoma, epithelial ovarian cancer, and pancreatic cancer, miR-381 acts as an antioncogene. Overexpression of miR-381 can lead to downregulation of those oncogenic signaling pathways or genes in oncogenic signaling axes. In primary glioma, miR-381 acts as an oncogene and modulates the PI3K/AKT and ERK pathways. However, under most conditions, miR-381 acts as an antioncogene. For example, in pancreatic cancer, the overexpression of miR-381 leads to reduced cell proliferation. Delivery of miR-381 is a potential clinical application (Table 1).

**TABLE 1:** MiR-381 participates in multiple signaling pathways.

Tumor type	Up/down regulation	Effects upon ectopic expression in cell lines	Target genes	Affected biological pathway/process	Oncogenic/suppressor role	References
Breast cancer	Down		CTNNB1, RhoA, ROCK1, c-MYC	Wnt pathway	Suppressor	(7)
Endometrial carcinoma	Down	Inhibit cell migration and invasion	IFG-1R	AKT/ERK pathway	Suppressor	(11)
Breast cancer	Down	Decrease cell proliferation	Nicotinamide phosphoribosyl transferase (NAMPT)	NAD salvage pathway	Suppressor	(12)
Glioma	Up	Promote cell proliferation	LRRC4	AKT/ERK pathway	Oncogenic	(19, 20)
Lung adenocarcinoma	Down	Decrease cell proliferation and invasion	LMO3	PI3K/AKT pathway	Suppressor	(9)
Pituitary tumor	Down		PTTG1	P53 pathway	Suppressor	(14)
Epithelial ovarian cancer	Down	Decrease cell proliferation and invasion	Yin Yang 1 (YY1)	Wnt pathway	Suppressor	(15)
Gastric cancer	Down	Decrease cell proliferation and invasion	ROCK2		Suppressor	(16)
Epithelioid sarcoma	Up	Promote cell invasion	SMARCB1	SMARCB1 pathway	Oncogenic	(17, 18)
Pancreatic cancer	Down	Decrease cell proliferation and invasion	ETS1	PI3K/AKT/mTOR pathway	Suppressor	(13)

## Biological Functions of miR-381 in Human Cancers

MiRNAs act as either oncogenes or tumor suppressors in various human cancers.

However, studies of microRNA-381 in cancer have revealed complex results. MiR-381 has been shown to play both oncogenic and antioncogenic roles via its involvement in EMT, cell cycle control, chemotherapeutic drug resistance, radioresistance, and immune responses. Here, we summarize the role of miR-381 in cellular pathways (Tables 2, 3).

**TABLE 2:** MiR-381 participates in epithelial–mesenchymal transition (EMT) processes.

Tumor type	Up/down regulation	Effects upon cell lines	Target genes	Affected biological pathway/process	Oncogenic/suppressor role	References
Oral squamous cell carcinoma	Down	Decrease cell proliferation and invasion	Fibroblast growth factor receptor 2 (FGFR2)	EMT	Suppressor	(21)
Breast cancer	Down	Decrease cell proliferation and invasion	C/EBPα	EMT	Suppressor	(22)
Pancreatic ductal adenocarcinoma	Down	Decrease cell proliferation and invasion	CXCR4	EMT	Suppressor	(23)
Breast cancer	Down	Decrease cell proliferation	CXCR4	EMT	Suppressor	(24)
Cervical cancer	Down	Decrease cell invasion	HOXA13	EMT	Suppressor	(25)
Cervical cancer	Down	Decrease cell proliferation and invasion	FGF7	EMT	Suppressor	(26)
Colorectal cancer	Down	Decrease cell proliferation and invasion	Twist 1	EMT	Suppressor	(27)
Gastric carcinoma	Down	Decrease cell proliferation and invasion	Sox4	EMT	Suppressor	(28)
Gastric carcinoma	Down	Decrease cell proliferation and invasion	CUL4B	EMT	Suppressor	(29)
Gastric carcinoma	Down	Decrease cell proliferation and invasion	TMEM16A	EMT	Suppressor	(30)
Gastric carcinoma	Down	Decrease cell proliferation and invasion	ZEB1	EMT	Suppressor	(31)
Osteosarcoma	Down	Decrease cell migration and invasion	ZEB1	EMT	Suppressor	(32)
Lung adenocarcinoma	Down	Decrease cell proliferation and invasion	Inhibitor of differentiation 1 (ID1)	EMT	Suppressor	(33)
Bladder cancer	Down	Decrease cell migration	MET and CCNA2	EMT	Suppressor	(34)
Colon cancer	Down	Cell proliferation	LRH-1	EMT	Suppressor	(35)
Hepatocellular carcinoma	Down	Decrease cell proliferation and invasion	LRH-1	EMT	Suppressor	(36)

**TABLE 3:** MiR-381 participated in multiple cellular processes.

Tumor type	Up/down regulation	Effects upon cell lines	Target genes	Biological processes	Oncogenic/suppressor role	References
Breast cancer	Down	Decrease cell proliferation and invasion	JARID1B	Inhibit cell cycle	Suppressor	(37)
Renal cancer	Down	Decrease cell proliferation	WEE1	Inhibit cell cycle	Suppressor	(38, 39)
Rectal carcinoma	Down	Decrease cell proliferation	UBE2C	Inhibit cell cycle	Suppressor	(40)
Breast cancer	Down	N/A	FYN	Drug sensitivity	Suppressor	(41)
Breast cancer	Down	N/A	MDR1	Drug sensitivity	Suppressor	(42)
Leukemia	Down	N/A	MDR1	Drug sensitivity	Suppressor	(43)
Renal carcinoma	Down	N/A	N/A	Drug sensitivity	Suppressor	(44)
NSCLC	Down	N/A	Inhibitor of differentiation 1 (ID1)	Drug sensitivity	Suppressor	(45)
Chondrosarcoma	Down	N/A	VEGF-C	Inhibit lymphangiogenesis	Suppressor	(46)
Hepatocellular	Down	N/A	VEGFA	Inhibit angiogenesis	Suppressor	(47)
Esophageal squamous cell carcinoma	Down	N/A	XIAP	Promote radiosensitivity	Suppressor	(48, 49)
NSCLC	Down	Inhibit cell migration	LRH-1	Inhibit metastasis	Suppressor	(50)
T cells	Up	N/A	CD1C	Inhibit CD1c, and promote IL-10	Suppressor	(51)
NSCLC	Down	Decrease cell proliferation and invasion	ADAR1	Promote innate immunity	Suppressor	(52)
Glioblastoma multiforme	Up	N/A	NEFL	Drug sensitivity	Oncogenic	(53)
Osteosarcoma	Up	N/A	LRRC4	Drug sensitivity	Oncogenic	(54)

### Role of miR-381 in Epithelial–Mesenchymal Transition

The transformation of epithelial cells to mesenchymal cells, also called EMT, is one of most important mechanisms in the development of many tumors. During this process, epithelial tumor cells lose cell-to-cell adhesion and acquire mesenchymal properties (55), thus acquiring invasion and migration abilities. At the molecular level, epithelial markers such as E-cadherin are downregulated, and mesenchymal markers such as N-cadherin are upregulated. Matrix metalloproteinase-2 (MMP-2) is highly expressed (56, 57). EMT has been found to be involved in multiple cancer processes, including migration and invasion, and especially in local invasion and metastasis of aggressive tumors (58).

MiR-381 directly targets fibroblast growth factor receptor 2 (FGFR2), leading to apoptosis and reduced cell proliferation. The expression of miR-381 is downregulated in oral squamous cell carcinoma (OSCC) tissues and cell lines. Overexpression of miR-381 in OSCC cell lines inhibits cell proliferation by increasing the proportion of G1/G0-phase cells, indicating that miR-381 acts as a tumor suppressor, possibly because miR-381 targets FGFR2 and downregulates it. FGFR2, a member of the FGF family, has been shown to be associated with several aspects of cancer progression, including cell proliferation, differentiation, and migration. In OSCC cells, FGFR2 suppression leads to reduced cell proliferation and increased apoptosis. Thus, by targeting FGFR2 and inhibiting EMT, miR-381-3p acts as a tumor suppressor (21).

MiR-381 can directly bind to the 3′UTR of CCAAT/enhancer-binding protein α (C/EBPα) and inhibit its expression. C/EBPα is a transcription factor that functions by binding to the AATTGTC sequence in the promoter region of the Cx43 gene. Thus, miR-381 inhibits Cx43 expression via the C/EBPα-binding element AATTGTC in the Cx43 promoter region. Cx43 is a vital regulator of metastasis and migration in multiple tumors. Thus, miR-381 can suppress the C/EBPα- and Cx43-dependent invasion of breast cancer cells (22).

However, the role of Cx43 remains controversial. On the one hand, Cx43 plays protective roles. Cx43 can sensitize NSCLC cells to chemotherapy by inhibiting EMT (59). The absence of Cx43 can enhance the aggressiveness of pancreatic cancer, indicating its protective effects (60). On the other hand, Cx43 has been reported to enhance the migration and invasion of tumor cells via p38 (61). In one study, Cx43 was highly expressed in aggressive breast cancer (22).

The transcription factor C/EBPα is considered to act as a tumor suppressor. C/EBPα was found to induce the transdifferentiation of B-cell lymphoma cells (62). However, C/EBPα can also function as a prognostic factor for poor survival in hepatocellular carcinoma (HCC) (63). In contrast, a study showed that C/EBPα acts as a tumor suppressor in metastatic breast cancer cells (22). Thus, the miR-381–C/EBPα–Cx43 axis might be a therapeutic target in metastatic breast cancer (61).

The long non-coding RNA DLEU1 is upregulated in multiple myeloma, leukemia, and pancreatic ductal adenocarcinoma (PDAC). Knockdown of DLEU1 in PDAC cell lines inhibits cell proliferation, invasion, and migration. MiR-381 is targeted by DLEU1 and targets the downstream protein CXCR4, a common chemokine receptor involved in infection and cancers. In addition, CXCR4 is an indicator of poor prognosis. The DLEU–miR-381–CXCR4 pathway may thus serve as a target in PDAC (23).

In another study, miR-381 expression was also found to be reduced in breast cancer compared with adjacent normal tissues. Overexpression of miR-381 in breast cancer cell lines reduces cell proliferation and reverses EMT, as evidenced by the increased level of E-cadherin (an epithelial marker) and decreased level of N-cadherin (mesenchymal marker) (55). Inhibition of miR-381 increases the expression of MMP-2 and MMP-9 (56, 57), indicating that downregulation of miR-381 leads to cancer cell migration and invasion. One study showed that miR-381 directly targets CXCR4, which is involved in cell migration and invasion. By targeting CXCR4, miR-381 plays an important role in breast cancer cell proliferation, EMT, and metastasis (24).

The long non-coding RNA DLEU1 is highly expressed in cervical cancer tissue and cervical cancer cell lines. Overexpression of DLEU1 in cervical cancer cell lines promotes cell proliferation and invasion. By sponging miR-381 in cervical cancer cells, DLEU1 inhibits miR-381, which directly targets HOXA13. HOXA13 has been found to be upregulated in gastric cancer and to enhance cancer cell invasion by promoting EMT progression (64). Thus, DLEU1 facilitates EMT, resulting in progression and invasion of cervical cancer via the DLEU1–miR-381–HOX13 axis (25).

Moreover, another group found that miR-381 is expressed at lower levels in cervical cancer tissues and cells than in their non-cancerous counterparts. Overexpression of miR-381 suppresses cervical cancer cell proliferation. FGF genes are members of a family of heparin-binding genes involved in multiple cellular processes, including growth, repair, migration, and invasion (65). FGF7, also called keratinocyte growth factor (KFG), is an epithelial cell-specific growth factor that is related to EMT progression (66, 67). MiR-381 targets FGF7 in cervical cancer cells and arrests the cell cycle at the G0/G1 transition, thus inhibiting cancer cell growth. By targeting FGF7, miR-381 also suppresses the migration and invasion of cervical cell lines (26).

MiR-381 is downregulated in colorectal cancer tissues. In colorectal cancer cell lines, overexpression of miR-381 can inhibit cell proliferation, migration, and invasion. Mechanistically, miR-381 directly targets Twist 1, a substantial inducer of EMT. Overexpression of Twist1 can attenuate the inhibitory effects of miR-381 on colorectal cancer cells, resulting in cell proliferation and invasion. Thus, via Twist1, miR-381 inhibits EMT, cell migration, and invasion (27).

MicroRNA 381 expression is downregulated in gastric carcinoma tissues. In gastric carcinoma cell lines, downregulation of miR-381 is related to cell migration and invasion. MicroRNA 381 targets Sox4, a master regulator of EMT in human cancers, and its expression. In conclusion, microRNA 381 can inhibit gastric carcinoma cell metastasis and EMT via suppressing Sox4 (28).

Another group found that Cullin 4B (CUL4B) is another crucial target of miR-381. CUL4B is a substrate component of the CRL4B ubiquitin ligase complex, playing an important role in ubiquitin-mediated proteolysis and tumorigenesis. CUL4B has been found to be upregulated in various cancers, such as lung, bladder, and colon cancers, thus contributing to cell proliferation, invasion, and the metastasis of malignant tumors. CUL4B epigenetically inhibits many tumor suppressors, including insulin-like growth factor-binding protein 3 (IGFBP3), PTEN, and p16. It is also involved in the Wnt/β-catenin signaling pathway. In gastric carcinoma, CUL4B is upregulated and targeted by miR-381 and miR-489, and silencing of CUL4B inhibits the proliferation and migration of gastric cancer cells via the Wnt/β-catenin signaling pathway. Wnt/β-catenin is related to EMT progression. Thus, via the miR-381/489–CUL4B axis, miR-381 and miR-489 suppress cell proliferation, gastric carcinoma invasion, and EMT (29).

Transmembrane protein 16A (TMEM16A), also called ANO1, DOG1, and TAOS2, is a calcium-activated chloride channel with various functions, such as sensory transduction and epithelial secretion. In addition, TMEM16A plays vital roles in tumorigenesis, tumor metastasis, cell proliferation, and apoptosis (68, 69) as a candidate oncogene. TMEM16A is highly regulated in gastric cancer and facilitates cell migration and invasion. MiR-381 directly targets TMEM16A and inhibits cell proliferation, invasion, and migration in gastric carcinoma cell lines. Moreover, overexpression of miR-381 inhibited tumor growth and metastasis in a nude mouse tumor model. Based on the results of a previous study showing that TMEM16A contributes to cell invasion via the TGF-β signaling pathway, miR-381 suppresses the TGF-β signaling pathway and, as a result, inhibits EMT. TGF-β is an important factor that can induce EMT, and TMEM16A can promote TGF-β secretion (70). Thus, by targeting TMEM16A, miR-381 can suppress the TGF-β signaling pathway and inhibit EMT progression, ultimately suppressing cell proliferation, migration, and invasion (30).

Another group found additional information about relationships of long non-coding RNAs with miR-381. The long non-coding RNA CAT104 is highly expressed in gastric carcinoma cell lines, and its expression is negatively correlated with miR-381 expression. In gastric carcinoma cell lines, knockdown of CAT104 has effects similar to those of an miR-381 inhibitor, indicating that CAT104 inhibits the expression of miR-381, resulting in enhanced cell migration and invasion. Furthermore, miR-381 targets ZEB1, which is involved in the Wnt/β-catenin pathway, and induces apoptosis. Thus, the CAT104–miR 381–ZEB1 pathway is an important pathway in gastric carcinoma cell migration and invasion (31).

In addition, the long non-coding RNA CAT104 is highly expressed in osteosarcoma cell lines. Knockdown of CAT104 in osteosarcoma cell lines inhibits cell migration and invasion. CAT104 targets MiR-381, and miR-381 targets ZEB1. ZEB1, a member of the ZEB family, is the key inducer of EMT progression. Overexpression of miR-381 inhibits the Wnt/β-catenin pathway. CAT104 exerts inhibitory effects through both the miR-381–ZEB1 pathway and the Wnt/β-catenin pathway. Thus, CAT104 and miR-381 could be novel targets in osteosarcoma (32).

MiR-381 is downregulated in lung adenocarcinoma. Inhibitor of differentiation 1 (ID1) expression is dependent on Src signaling. Src, a member of the src family, is a component of the focal adhesion kinase complex and is involved in cell adhesion and migration. The Src signaling pathway contributes to cell cycle control and angiogenesis. A previous study showed that src kinases are involved in lung cancer cell migration via SMAD and ID1.

Inhibitor of differentiation 1 belongs to the helix–loop–helix protein family, and ID proteins are essential proliferative factors in many cell lines. Bone morphogenic proteins (BMPs), transforming growth factor-β-related growth factors, regulate ID family proteins through Smad transcription factors. ID1 plays an important role in tumor cell differentiation and proliferation, as well as in tumor angiogenesis, and metastasis. In cancer, ID1 is associated with an aggressive phenotype. In addition, inhibition of ID1 decreases tumor growth in animal models. A previous study reported that ID1 is overexpressed in lung adenocarcinoma and that ID1 expression correlates with that of Src and MMP9, which are associated with EMT. Another study showed that ID1 expression could be an independent prognostic factor in lung adenocarcinoma patients. MiR-381 is decreased in lung adenocarcinoma tissues, and miR-381 binds to ID1 and inhibits its expression. Src inhibition can lead to increased miR-381 expression and ID1 downregulation. Overexpression of miR-381 leads to suppressed ID1 expression and inhibits cell migration and invasion. Thus, miR-381 is involved in the Src–ID1 pathway and may be a potential target via EMT inhibition (33).

Overexpression of miR-381-3p significantly inhibits cell proliferation and migration.

MiR-381 inhibits bladder cancer cell proliferation by regulating CDK6, resulting in G1-phase arrest. MiR-381 can also inhibit migration by downregulating MET and CCNA2, thus resulting in EMT progression (34).

In colon cancer, miR-381 is significantly downregulated. Inhibition of miR-381 results in cell proliferation, and liver receptor homolog-1 (LRH-1) is the target of miR-381. Thus, LRH-1, a member of the nuclear receptor family of regulatory transcription factors, could be an important oncogene involved in tumor invasion. LRH-1 is highly expressed in gastric cancer and breast cancer (71, 72), and it plays an important role in the Wnt/β-catenin pathway, which suppresses immune activation and EMT (72). In conclusion, through LRH-1, miR-381 inhibits tumor progression in colon cancer (35).

In hepatocellular carcinoma tissues, miR-381 is downregulated, and overexpression of miR-381 in hepatocellular carcinoma cell lines results in decreased cell proliferation and invasion. MiR-381 directly targets LRH-1, which is involved in the development of multiple cancers. LRH-1 is overexpressed in breast cancer and acts as an estrogen receptor target gene. LRH-1 regulates estrogen receptor expression, which promotes cell proliferation in breast cancer. Overexpression of LRH-1 in breast cancer results in cell migration and invasion. LRH-1 is also overexpressed in pancreatic cancer, colon cancer, and gastric cancer, indicating that it is a potential oncogenic factor. Moreover, LRH-1 is an activator of the Wnt signaling pathway. By inducing cyclin E1 expression, LRH-1 is involved in the Wnt signaling pathway, which is associated with cancer metastasis. In one study, miR-381 was shown to target LRH-1 and suppress the Wnt signaling pathway, ultimately inhibiting cell growth and invasion (36) (Table 2).

### Cell Cycle Control

MiR-381 also plays an important role in cell cycle control, including exerting such effects as increasing the proportion of cells in G1 phase and inhibiting the G2/M transition, resulting in *decreased* cell proliferation during cancer development.

Ivano Mocavini et al. performed a computational analysis of cancer tissues from 103 breast cancer patients and similarly concluded that the levels of miR-381 and miR-486 are reduced in cancer tissues (73). Histone tail modifications play an important role in regulating chromatin structure, and histone methylation is a key process in histone tail modification (74). Methylation marks can be erased by specific histone demethylases (HDMs), which can be divided into two families, the LSD1 family and the Jumonji C-domain-containing (JHDM) family. The level of JARID1A, a member of the JHDM family (75), has been found to be increased in gastric cancer and cervical cancer, and it is considered a potential target (76, 77). JARID1B, in parallel to JARID1A, is increased in breast cancer, lung cancer, and melanomas (37, 78–80). Both miR-381 and miR-486 can target JARID1B and inhibit its expression, which can induce DNA damage accumulation. Overexpression of miR-381 and miR-486, which is induced by upregulated BRCA1 expression, can increase the proportion of G1/G0-phase cells. BRCA1 is a crucial factor involved in breast cancer metastasis. Thus, miR-381-induced JARID1B downregulation causes hypersensitivity to DNA damage and BRCA1 upregulation, which plays an important role in breast cancer migration (73).

MiR-381 is a crucial regulatory factor for WEE1, which is involved in DNA duplication and chromosome condensation. WEE1 depletion blocks S phase completion. WEE1 catalyzes phosphorylation and is an inhibitor of the G2/M transition. On the other hand, by promoting inhibitory phosphorylation of cyclin-dependent kinases (CDKs), WEE1 overexpression causes G2 arrest. Thus, WEE1 inhibition promotes antitumor effects. Regulation of CDKs by WEE1 is found in various cancers, including glioblastoma, lung carcinoma, and breast cancer. Thus, the miR-381–WEE1–cell cycle pathway can be a potential therapeutic target for suppressing tumor growth (38, 39).

UBE2C encodes a member of the E2 ubiquitin-conjugating enzyme family that can promote mitotic exit and cell cycle progression. Recently, some studies have shown that UBE2C is involved in the progression of various cancers, including glioma, hepatocellular carcinoma, gastric cancer, breast cancer, and non-small cell lung cancer (NSCLC). A previous study detected a higher expression of UBE2C in advanced colon cancer. In addition, UBE2C is more highly expressed in rectal carcinoma tissues than in the corresponding non-cancerous tissues and promotes cell proliferation and invasion. *In vivo*, a study in a xenograft mouse model showed that UBE2C promotes tumor growth, suggesting that UBE2C promotes cell proliferation, in rectal carcinoma. MiR-381 can directly bind to UBE2C and inhibit its expression. In a xenograft mouse model, overexpression of miR-381 resulted in tumor growth inhibition, indicating that miR-381 could be a future potential therapeutic target (40).

### Drug Resistance

Drug resistance has historically been a major problem in cancer chemotherapy. MiR-381 can sensitize cells to chemotherapeutic drugs via multiple mechanisms, including inhibiting the expression of drug resistance genes and blocking cell signaling pathways.

Doxorubicin (DOX) is a common first-line chemotherapeutic agent for breast cancer, and DOX resistance remains a major challenge. In DOX-resistant breast cancer cells, miR-381 is downregulated. FYN, which is involved in the MAPK pathway, is suppressed by the binding of miR-381 to its 3′UTR. The MAPK signaling pathway is involved in cell proliferation, differentiation, and survival; apoptosis; immunity; and drug resistance (81, 82). In addition, the MAPK pathway is associated with resistance to drugs, including DOX (83–85). Thus, miR-381 can suppress the FYN–MAPK pathway. Overexpression of miR-381 can increase DOX sensitivity in breast cancer cell lines. Thus, miR-381 increases drug sensitivity in breast cancer via the FYN–MAPK pathway (41).

Cisplatin (DDP), another drug to which breast cancer is commonly resistant, is also related to miR-381. MiR-381 expression is decreased in cisplatin-resistant breast cancer tissues and cell lines, and miR-381 overexpression can increase the sensitivity of breast cancer cells to cisplatin. Similar to its effect on DOX sensitivity, miR-381 targets MDR1 and promotes cisplatin sensitivity in breast cancer cells (42). MDR1 is a member of the ATP-binding cassette (ABC) transporter superfamily. It is associated with resistance to multiple drugs in various cancers (86–88). Genetic knockdown of MDR1 enhances the chemosensitivity of breast cancer cells (89). Thus, targeting of MDR1 by miR-381 could be a therapeutic strategy (42).

Multidrug resistance (MDR) is a main cause of chemotherapeutic failure. One of most common mechanisms is the efflux of hydrophobic drugs. P-glycoprotein (P-gp), an ABC transporter, plays an important role in this process. Encoded by the MDR1 gene (also called ABCB1), p-gp is widely expressed in the gastrointestinal tract, liver, and kidneys. In addition, it is overexpressed in many cancers, and p-gp overexpression leads to multidrug resistance in some cancers, such as leukemia. In p-gp-overexpressing leukemia cell lines, the levels of miR-381 and miR-495 are reduced. Both miR-381 and miR-495 target the 3′UTR of the MDR1 gene and inhibit its expression. Restoring the expression of miR-381 results in suppressed expression of the MDR1 gene and its protein, P-gp, thus increasing drug uptake by leukemia cells. Thus, miR-381 and miR-495 provide a novel approach to leukemia therapy (43).

In clear cell renal cell carcinoma tissues, the expression of miR-381 is decreased compared to that in normal tissues. Overexpression of miR-381 in clear cell renal cell carcinoma cell lines can enhance the antitumor effects of cisplatin (Ci) and paclitaxel (Pa). In contrast, inhibition of miR-381 in these cell lines can promote cell proliferation. In a mouse model, cells with miR-381 inhibition were injected into nude mice, and inhibition of miR-381 was found to promote chemoresistance to Ci and Pa (44).

Drug resistance is also a major problem in NSCLC. NF-κB is a key modulator of cell death via transactivation of antiapoptotic genes and mediates cellular resistance to cisplatin-induced apoptosis. In NSCLC cell lines, activation of NF-κB leads to cisplatin chemoresistance, and inhibition of NF-κB can enhance cisplatin efficiency. MiR-381 suppresses the activation of NF-κB via ID1. Overexpression of miR-381 inhibits the proliferation and colony-forming ability of NSCLC cells. Furthermore, overexpression of miR-381 induces an increase in the proportion of G0/G1-phase cells and a decrease in the proportion of S-phase cells. Moreover, delivery of miR-381 decreases the IC50 values of cisplatin by six fold in NSCLC cell lines, indicating that miR-381 induces cisplatin sensitivity in NSCLC cells. Thus, in NSCLC cells, miR-381 regulates the cell cycle and chemosensitivity, and restoration of miR-381 expression may be a therapeutic approach in NSCLC (45).

Though in most occasions miR-381 exerts tumor suppressor functions, in some tumors, miR-381 exerts oncogenic functions.

Glioblastoma multiforme (GBM) is one of the most malignant gliomas. Temozolomide (TMZ) has been widely used in GBM patients. However, resistance to TMZ is a major obstacle to the success of GBM chemotherapy. The filament light polypeptide (NEFL) gene, a tumor suppressor gene, is also a target of miR-381. The NEFL gene, located on chromosome 8p21, encodes a type IV filament that functions in maintaining neuronal caliber and transporting neurotransmitters to axons and other dendrites. NEFL acts as a tumor suppressor gene in various cancers, including breast cancer and head and neck cancer. In glioma tissues, NEFL expression is reduced. Overexpression of NEFL inhibits cell proliferation and invasion. In addition, overexpression of NEFL increases the chemosensitivity of glioma cells to TMZ treatment by downregulating the multidrug resistance factors ABCG2, ABCC3, and ABCC5, which are ABC transporters and function as xenobiotic transporters. MiR-381 interacts with NEFL and inhibits its expression. In conclusion, miR-381 regulates the chemosensitivity of glioma cell lines to TMZ treatment via NEFL. Downregulation of miR-381 enhances the sensitivity of glioblastoma cells to TMZ by reducing NEFL expression. Thus, the miR-381–NEFL axis is critical for TMZ resistance in GBM and may be a therapeutic target in glioma (53).

Osteosarcoma (OS) is the most common malignant bone tumor. In osteosarcoma cell lines, overexpression of miR-381 results in proliferation and invasion. As noted earlier, LRRC4 is a direct target gene of miR-381. Chemotherapeutic drugs such as cisplatin and DOX are widely used in the treatment of OS. Drug resistance is the main reason for chemotherapeutic failure. Inhibition of miR-381 and overexpression of LRRC4 enhance the sensitivity in osteosarcoma cell lines to cisplatin. Consequently, inhibition of miR-381 and overexpression of LRRC4 result in inhibited expression of multidrug resistance genes, such as ABCC1, ABCC3, and ABCG2. A further study showed that LRRC4 overexpression decreases the expression of p70S6K, a critical downstream substrate known as an indicator of the mTOR pathway activity. The mTOR pathway is commonly involved in cancer. Thus, miR-381 could act as a target via its involvement in the mTOR pathway (54).

### Lymphangiogenesis

Tumor metastasis is associated with many processes, including proliferation, migration, invasion, angiogenesis, and lymphangiogenesis (90–92). Lymphangiogenesis is a key step in cancer metastasis. VEGF-C is a key factor in lymphangiogenesis and plays a vital role in lymphangiogenesis and lymphatic metastasis. VEGF-C plays an important role in various human cancers (93–96).

Basic fibroblast growth factor (bFGF)/FGF-2 encode proteins associated with proliferation. These proteins are associated with progression in many cancers and are also associated with lymph node metastasis and prognosis. Our group previously reported that bFGF increases VEGF-A expression and promotes angiogenesis (97), and bFGF was shown to be involved in metastatic chondrosarcoma. bFGF expression is increased in chondrosarcoma (97), and VEGF-C expression is higher in tumor specimens than in normal tissues. Moreover, expression of bFGF is associated with VEGF-C expression in human chondrosarcoma. Specifically, bFGF promotes VEGF-C expression via the PDGFR–c-Src pathway in chondrosarcoma. MiR-381 can bind to the 3′UTR of the VEGF-C gene and repress its expression, thus repressing VEGF-C-mediated lymphangiogenesis. Furthermore, PDGFR and c-Src inhibitors reverse miR-381 expression and VEGF-C activity, indicating that the PDGFR–c-Src pathway is upstream of bFGF in regulating miR-381 expression. In conclusion, bFGF can enhance VEGF-C by downregulating miR-381 expression via the PDGFR–c-Src pathway, resulting in lymphangiogenesis in chondrosarcoma. MiR-381, as a link, could be a crucial target in lymphangiogenesis (46).

In tumor thrombi from hepatocellular carcinoma with portal vein tumor thrombosis (PVTT), miR-381 is downregulated. However, its target, VEGFA, is upregulated. VEGFA plays an important role in angiogenesis during tumor growth, and overexpression of miR-381 promotes cell proliferation. Thus, miR-381 and VEGFA may be involved in PVTT development (47).

### Radioresistance

Radioresistance has historically been a main reason for therapeutic failure in esophageal squamous cell carcinoma (ESCC). The mechanism of radioresistance is complicated and involves many molecular pathways. MiRNAs have been found to be associated with radioresistance. For example, the expression levels of microRNA 381 is lower in recurrent esophageal squamous cell carcinoma *in situ* after radiotherapy has than in primary esophageal squamous cell carcinoma. In addition, in an assay of six different esophageal squamous cell carcinoma cell lines, microRNA 381 expression was 2.5-fold higher in the most radiosensitive cell lines than in the least radiosensitive cell lines. Overexpression of miRNA-381 in esophageal squamous cell carcinoma cells increases their radiosensitivity and reduces their proliferation ability, indicating the negative role of miRNA-381 in esophageal squamous cell carcinoma. Inhibition of miRNA-381 leads to radioresistance and aggressive behaviors in esophageal squamous cell carcinoma cell lines. *In vivo*, cell lines with higher miRNA-381 expression levels generate tumors less aggressively, supporting the hypothesis that miRNA-381 inhibits tumor growth and aggressive behaviors. One study showed that miRNA-381 is associated with radiosensitivity in esophageal squamous cell carcinoma; miRNA-381 was increased in radiosensitive but decreased in radioresistant esophageal squamous cell carcinoma cell lines. *In vitro* studies further confirmed that overexpression of miRNA-381 increases the radiosensitivity and reduces the aggressive behavior of esophageal squamous cell carcinoma cells. MiRNA-381 sensitizes esophageal squamous cell carcinoma cells to irradiation and inhibits their proliferation, migration, and invasion, indicating that miRNA-381 is a potential biomarker and potential therapeutic target for esophageal squamous cell carcinoma (48).

Moreover, another group found that miR-381 expression is downregulated in esophageal squamous cell carcinoma tissues and cells, and that restoration of miR-381 expression enhances the apoptosis of esophageal squamous cell carcinoma cells. XIAP is an inhibitor of apoptotic cell death. By targeting XIAP, which is upregulated in esophageal squamous cell carcinoma tissues, miR-381 induces caspase-3-dependent apoptosis in these cells. This result indicates that enhancing miR-381 could be a novel approach to restore radiosensitivity in esophageal squamous cell carcinoma by targeting XIAP (49).

MiR-381 expression is decreased in NSCLC tissues and cell lines. *In vitro*, overexpression of miR-381 inhibits cell migration. *In vivo*, overexpression of miR-381 reduces lung cancer metastasis. LRH-1 is a direct target of miR-381. Thus, in NSCLC, miR-381 plays an important role in tumor metastasis and can be a novel prognostic biomarker and therapeutic target (50).

### Immune Responses

The CD1 molecule plays an important role in antigen presentation. Members of the CD1 family are MHC-like glycoproteins, and the CD1 family can be divided into classes I, II, and III according to gene homology. Activated T cells, including invariant natural killer T (NKT) and CD1-restricted ab T cells, can recognize CD1 and the presented antigen. CD1c is a member of the class I CD1 family. By binding to the 3′UTR of CD1c, miR-381 significantly reduces CD1c expression at both the mRNA and protein levels. Treatment with an miR-381 inhibitor was found to increase the level of CD1c and enhance CD1c-mediated T-cell immune responses. During differentiation from monocytes to dendritic cells (DCs), IL-10, an immune suppressor, greatly suppresses CD1c expression. MiR-381 can inhibit CD1c expression via IL-10 (51).

Adenosine deaminase acting on RNA (ADAR), is an RNA polymerase that converts adenosine (A) to inosine (I). Humans express three ADARs: ADAR1, ADAR2, and ADAR3. The conversion of A to I by ADAR results in cancer development. ADAR1 upregulation has been reported to be involved in hepatocellular carcinoma development. In addition, ADAR1 was shown to be a candidate oncogene. A recent study showed that ADAR1 is a suppressor of dsRNA-triggered induction and action of interferon, which results in inhibition of innate immunity (98). In NSCLC cell lines, ADAR1 is overexpressed. In an ADAR1 knockdown mouse tumor model, the tumors were smaller than those in control mice. In NSCLC cell lines, ADAR1 overexpression results in cell proliferation and invasion. ADAR1 targets miR-381 and is involved in cancer development (52).

MiR-381 plays different roles in cancer development across different types of cancer. In OSCC, miR-381 suppresses EMT by inhibiting FGFR2. However, the role of miR-381 is controversial. On the one hand, miR-381 inhibits Cx43 and CXCR4, which can regulate metastasis and cell migration in breast cancer. On the other hand, Cx43 can sensitize NSCLC cells to chemotherapy. In cervical cancer, miR-381 inhibits HOXA13 and FGF7, which can facilitate invasion and migration. In gastric carcinoma, miR-381 inhibits Sox4, TMEM16A, and ZEB1 and suppresses metastasis. In lung adenocarcinoma, miR-381 inhibits ID1 and suppresses EMT. In colon cancer and hepatocellular carcinoma, miR-381 inhibits LRH-1 and suppresses cell growth. EMT is one of the most essential mechanisms in cancers, and via EMT, tumor cells can migrate and invade. Under most conditions, miR-381 acts as an inhibitor of EMT. Thus, overexpression of miR-381 inhibits cell proliferation and invasion.

MiR-381 is also involved in cell cycle control. In breast cancer, miR-381 inhibits WEE1 and blocks the G2/M transition. In colon cancer, miR-381 inhibits UBE2C and, as a result, suppresses cell growth. Moreover, miR-381 mediates drug resistance. In breast cancer, miR-381 increases sensitivity to DOX and cisplatin. MiR-381 can also increase cisplatin sensitivity in NSCLC. However, in GBM, downregulation of miR-381 increases TMZ sensitivity. Similar results have been observed in osteosarcoma; downregulation of miR-381 can inhibit the expression of multidrug resistance genes. In chondrosarcoma, miR-381 inhibits VEGF-dependent lymphangiogenesis, and in esophageal squamous cell carcinoma, miR-381 increases radiosensitivity. In NSCLC, miR-381 suppresses the immune response. The role of miR-381 is thus complicated and dependent on the type of cancer. MiR-381 has been proven to be involved in multiple processes, including EMT, cell cycle control, drug resistance, lymphangiogenesis, and radiosensitivity. However, under most conditions, miR-381 acts as an inhibitor of tumorigenesis. Delivery of miRNA to inhibit the process of tumorigenesis is a potential future direction. Considering that miR-381 can enhance chemo- and radiosensitivity, combination treatment with miR-381 and chemotherapy or radiation may be a novel and comprehensive therapeutic strategy for cancer.

## Clinical Applications

### MiR-381 Could Be a Novel Prognostic Biomarker

The current methods for diagnosing cancer, including detection of serum tumor markers, such as CA125, CA199, and CEA, and traditional imaging techniques, such as CT, have low sensitivity and specificity. MiRNAs can also be detected in serum or tissue samples; thus, miRNAs have potential use as biomarkers.

The levels of miR-381 were assessed in multiple cancers, and studies have shown that in some cancers, miR-381 is associated with prognosis and is thus a novel biomarker (Table 4). MiR-381 expression is decreased and C/EBPα expression is upregulated in aggressive breast cancer cells compared with non-aggressive breast cancer cells and tissues. Similarly, C/EBPα expression is higher, and miR-381 expression is lower in metastatic breast cancer tissues than in non-metastatic breast cancer tissues. Thus, miR-381 is a potential target and prognostic factor. In another study that enrolled 103 breast cancer patients, miR-381 was expressed at lower levels in breast cancer samples than in normal tissues. The Kaplan–Meier curve showed that patients with higher expression of miR-381 have longer survival times (*P* = 0.05) than those with lower expression (22). A similar study with 46 breast cancer patients showed similar results. Patients with lower levels of miR-381 had poorer survival (*P* = 0.023) than those with high levels of miR-381 (22). In addition, miR-381 is downregulated in colon cancer tissues compared with adjacent tissues and is even more strongly downregulated in stage III/IV compared with stage I/II colon cancer tissues (35). In colorectal cancer, miR-381 expression is decreased compared with that in paired normal mucosal tissue. A study involving 113 colorectal cancer patients showed that miR-381 downregulation is related to distant metastasis (*P* = 0.07) and a high TNM stage (*P* = 0.035) (27). In endometrial carcinoma, miR-381 expression is decreased. Analysis of 45 paired EC tissues and corresponding normal tissues showed that patients with lower levels of miR-381 expression had a higher FIGO stage (*P* = 0.001) and higher rates of lymph node metastasis (*P* = 0.004) and invasion (*P* = 0.021) than those with higher levels (11). In esophageal squamous cell carcinoma tissues, miR-381 expression is downregulated. Moreover, the expression level of microRNA 381 is lower in recurrent esophageal squamous cell carcinoma *in situ* after radiotherapy than in primary esophageal squamous cell carcinoma (49). In gastric cancer tissues, miR-381 expression is downregulated. Analysis of 60 paired gastric cancer tissues and tumor adjacent tissues showed that the expression of miR-381 in tumors with metastasis was lower than that in tumors without metastasis (*P* < 0.05). In addition, lower expression of miR-381 was found to be associated with lymphatic metastasis (*P* = 0.031) and a higher TNM stage (III + IV, *P* = 0.003) (28). Another study involving 20 patients also showed that miR-381 expression is downregulated in gastric cancer tissues compared with non-cancerous control tissues (29). Qinghua Cao et al. divided 103 gastric cancer patients into two groups and found that lower miR-381 expression was associated with lymph node metastasis (*P* = 0.002) and more advanced tumor stage (*P* = 0.000) (68). In glioma, the expression of miR-381 gradually increases with increasing WHO grade from I to IV. Total RNA was extracted from peripheral blood samples from 32 healthy individuals and 54 brain cancer patients. The expression of miR-381 in the peripheral blood of the brain cancer patients was significantly higher than that in the healthy individuals (*P* < 0.05). Thus, peripheral blood miR-381 can be used as a biomarker (20). In lung adenocarcinoma, a study involving 18 patients showed that miR-381 expression is correlated with event-free survival (EFS) (*P* = 0.003) and overall survival (OS) (*P* = 0.02) (33). In NSCLC, a study involving 124 patients showed that miR-381 expression is significantly associated with advanced TNM stage (*P* = 0.006) and lymph node metastasis (*P* = 0.001). Reduced miR-381 expression is related to poor prognostic characteristics. Moreover, the Kaplan–Meier curve showed that patients with higher miR-381 expression exhibited better overall survival (*P* = 0.0001), and miR-381 is an independent prognostic factor for 5-year survival (*P* = 0.002) (50). In a study of osteosarcoma, 60 patients were divided into two groups according to miR-381 expression, and miR-381 expression was found to be associated with survival time. Moreover, Kaplan–Meier analysis showed that patients with lower miR-381 expression had longer survival times (54). The expression levels of miR-381 in samples from primary EOC tumor tissues (*n* = 40) and normal ovarian epithelial tissues (*n* = 20) from endometrioid ovarian cancer (EOC) patients were compared, and the expression of miR-381 was lower in EOC tissues (*P* < 0.001). Reduced miR-381 levels are associated with higher tumor stage (*P* = 0.022), higher grade (*p* = 0.008), and lymph node metastasis (*p* = 0.077). MiR-381 expression in clear cell ovarian carcinomas (CCOC, *n* = 9), high-grade serous ovarian carcinomas (HGSCs, *n* = 12), and ovarian surface epithelial cells (OSES, *n* = 9) was analyzed by microarray profiling. MiR-381 was found to be reduced in CCOC and HGSC tissues (15).

**TABLE 4:** The relationship between miR-381 and various types of cancers’ prognosis.

Types of cancer	Expression level of miR-381	Overall survival	Metastasis	Tumor stage	References
Breast cancer	Lower in patients	Higher expression in longer survival patients	N/A	N/A	(22)
Colon cancer	Lower in patients	N/A	N/A	Lower expression in higher stage	(35)
Colorectal cancer	Lower in patients	N/A	Lower expression in distant metastasis	Lower expression in higher stage	(8)
Endometrial carcinoma	Lower in patients	N/A	Lower expression in lymph node metastasis	Lower expression in higher stage	(11)
Esophageal squamous cell carcinoma	Lower in patients	N/A	Lower expression in lymph node metastasis	Lower expression in higher stage	(49)
Gastric cancer	Lower in patients	N/A	Lower expression in lymph node metastasis	Lower expression in higher stage	(28, 29, 68)
Glioma	Higher in patients	N/A	N/A	Higher expression in higher stage	(20)
Lung adenocarcinoma	Lower in patients	Higher expression in longer survival patients	N/A	N/A	(33)
Non-small cell lung cancer	Lower in patients	Higher expression in longer survival patients	Lower expression in lymph node metastasis	Lower expression in higher stage	(50)
OS	Lower in patients	Higher expression in longer survival patients	N/A	N/A	(54)
Epithelial ovarian cancer	Lower in patients	N/A	Lower expression in lymph node metastasis	Lower expression in higher stage	(15)
Prostate cancer	Lower in patients	N/A	Lower expression in lymph node metastasis	Lower expression in higher stage	(99)

A decade ago, 40 miRNAs located in the 14q32 domain were identified as a large cluster. The 14q32 domain, also named Dlk1-Gtl2 in mice and Dlk-Dio3 in humans, plays an important role in many cancers, including melanoma, osteosarcoma, hepatocellular carcinoma, gastric cancer, ovarian cancer, and glioma. The expression of miRNAs in the 14q32.31 cluster, including miR-134, miR-154, miR-299, and so forth, is downregulated in human prostate cancer. Low expression levels of miRNAs in the 14q32.31 miRNA cluster are associated with high expression of PSA, an increased incidence of metastatic events, and lymph node invasion. The 14q32.31 miRNA cluster could thus be a prognostic factor in prostate cancer (99).

Although various studies have revealed that miR-381 is associated with cancer prognosis, miRNAs still have some disadvantages. The concentration of miRNA in the serum is too low to detect, which is a major clinical limitation. Detecting miRNAs in tissue can be cumbersome and time consuming. Further studies are needed to overcome the obstacles limiting the clinical application of miRNAs (50).

### Perspective on the Potential Therapeutic Role of miR-381 in Cancer

MiR-381 can act as a tumor suppressor in various cancers and, in contrast, can act as an oncogene in other types of cancers. In most studies, the detection of miR-381 is performed in tissue samples or in cell lines, and the expression of miR-381 is regulated by miRNA mimics or other inhibitors. However, these approaches cannot be used directly in cancer patients because RNase is widely present in serum and efficiently degrades miRNAs. Another problem is that miR-381 is restricted to the cytoplasm, which makes delivery of agents particularly difficult. Some strategies have been developed, and designing efficient delivery systems is the most common strategy. Nanoparticles are one of the most practical carriers (100). However, clinical application of miR-381-related agents has not been explored. The delivery of miR-381 agents is a major obstacle to be surmounted, and potential toxicities are another major problem for drug design. The present studies are insufficient and have not explored strategies to solve the major problems facing the clinical use of miRNAs—the delivery of miRNAs and the prevention of miRNA degradation and toxicity.

## Conclusion

MiR-381 plays important roles in tumor initiation, progression, and metastasis. The mechanism of miR-381 is complicated and remains to be further clarified. Various studies have been performed to explain the signaling pathways affected by miR-381 in cancer, and the findings have indicated that miR-381 could be a novel therapeutic target in cancer. In addition, clinical studies have revealed that miR-381 could be a biomarker to predict survival and prognosis in various cancers. However, the application of miRNAs in clinical cancer treatment still faces obstacles. More investigations are needed to explore the clinical applications of miR-381, which might become a potent therapeutic agent.

## Author Contributions

TZ contributed to the study conception and design. ZC and XZ wrote the main manuscript text and prepared the figures and tables. WL and LZ provided advice regarding the manuscript. All authors reviewed the manuscript.

## Conflict of Interest

The authors declare that the research was conducted in the absence of any commercial or financial relationships that could be construed as a potential conflict of interest.
